# Supplementary material for: Experimental and Numerical Study of Pd/Ta and PdCu/Ta Composites for Thermocatalytic Hydrogen Permeation
Source: Membranes (Basel). 2022 Dec 24;13(1):23. doi: 10.3390/membranes13010023 (PMC9863407; doi:10.3390/membranes13010023)
Supplement: Supplementary file 1 [file membranes-13-00023-s001.zip › membranes-2094909-supplementary.pdf]

membranes

MDPI

Supporting Inforamtion

Experimental and numerical study of Pd/Ta and PdCu/Ta composites for thermocatalytic hydrogen permeation

Seungbo Ryu [1,†], Arash Badakhsh [1,2,†], Jegyu Oh [3], Hyungchul Ham [3], Hyuntae Sohn [1,4], Sungpil Yoon [1] and Sunhee Choi [1,4,*]

1 Center for Hydrogen·Fuel Cell Research, Korea Institute of Science and Technology (KIST), Seoul 02792, Republic of Korea
2 PNDC, University of Strathclyde, Glasgow G68 0EF, UK
3 Department of Chemical Engineering, Inha University, Incheon 22212, Republic of Korea
4 Department of Energy and Environmental Engineering, KIST School, University of Science and Technology (UST), Seoul 02792, Republic of Korea
* Correspondence: shchoi@kist.re.kr; Tel.: +82-10-9256-5908; Fax: +82-2-958-5199
† These authors contributed equally to this work.

Membrane Preparation Process

Figure S1. illustrates the stepwise preparation process of membranes. DI denotes deionized water.

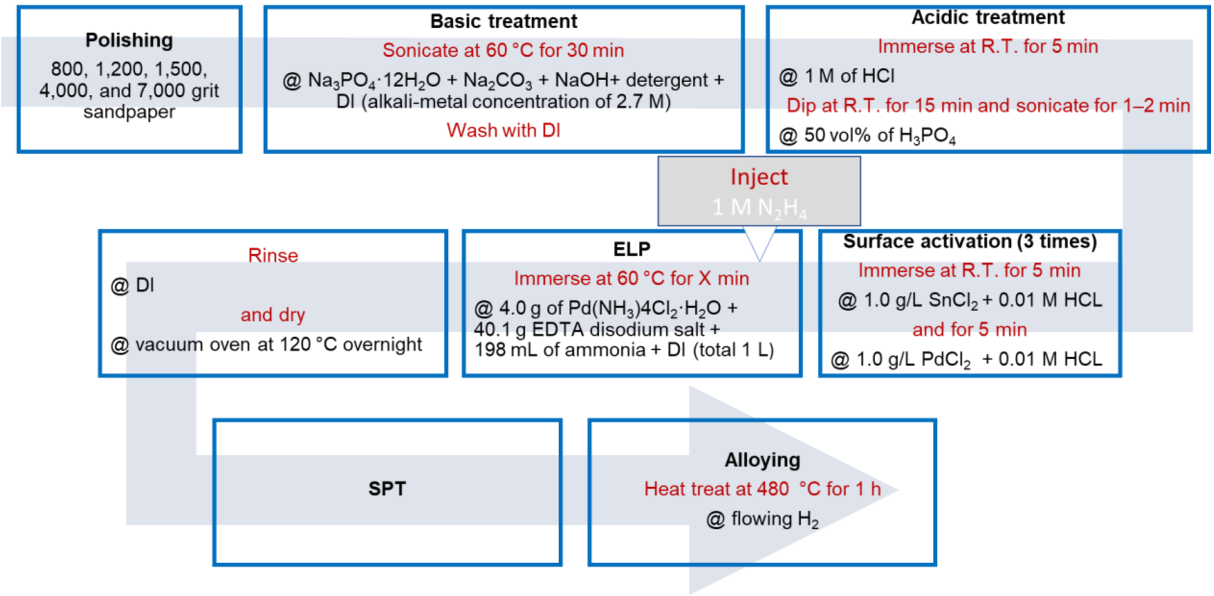

Figure S1. Stepwise preparation process of membranes using Ta support.

Sn Residue of the Pd/Ta Membrane Prepared by ELP

The Sn residue of the membranes prepared by ELP was analyzed using transmission electron microscopy (TEM, Talos TEM, Thermo Fisher Scientific). To this end, TEM was used to determine the threshold of the lowest amount of Sn, which other methods cannot capture. First, the deposited layer of the membrane was ground. Then, the ground powder was soaked in ethanol and dispersed on a copper grid.

At ~25 keV, a clear Sn signal is observed, so we can conclude that many Sn atoms remained, as shown in Figure S2c. Although Sn does not exist in all parts, it has been detected in the compound. This small number of residues can reduce the adhesion strength of the interface between Ta and Pd. Therefore, this new challenge should be addressed for long-term permeation operations. Previous studies have reported that Sn residues in Pd hydrogen separation membranes electroless plated over alumina decreased

selectivity [1]. In this work, where Pd is deposited on Ta, the performance also decreases due to the effect of Sn on the interface between Ta and Pd. The melting point of Sn is 231.9 °C, which is much lower than Ta's and Pd's melting points, *i.e.*, 3,017 °C and 1,555 °C, respectively. Thus, the phase of Sn easily changes with temperature, resulting in the inter-diffusion into other metals over time. While this diffusion is expected to enhance the stability by promoting alloys in binary systems (*e.g.*, PdCu), the effect in this tertiary system (Pd, Ta, and small amounts of Sn) has not yet been reported.

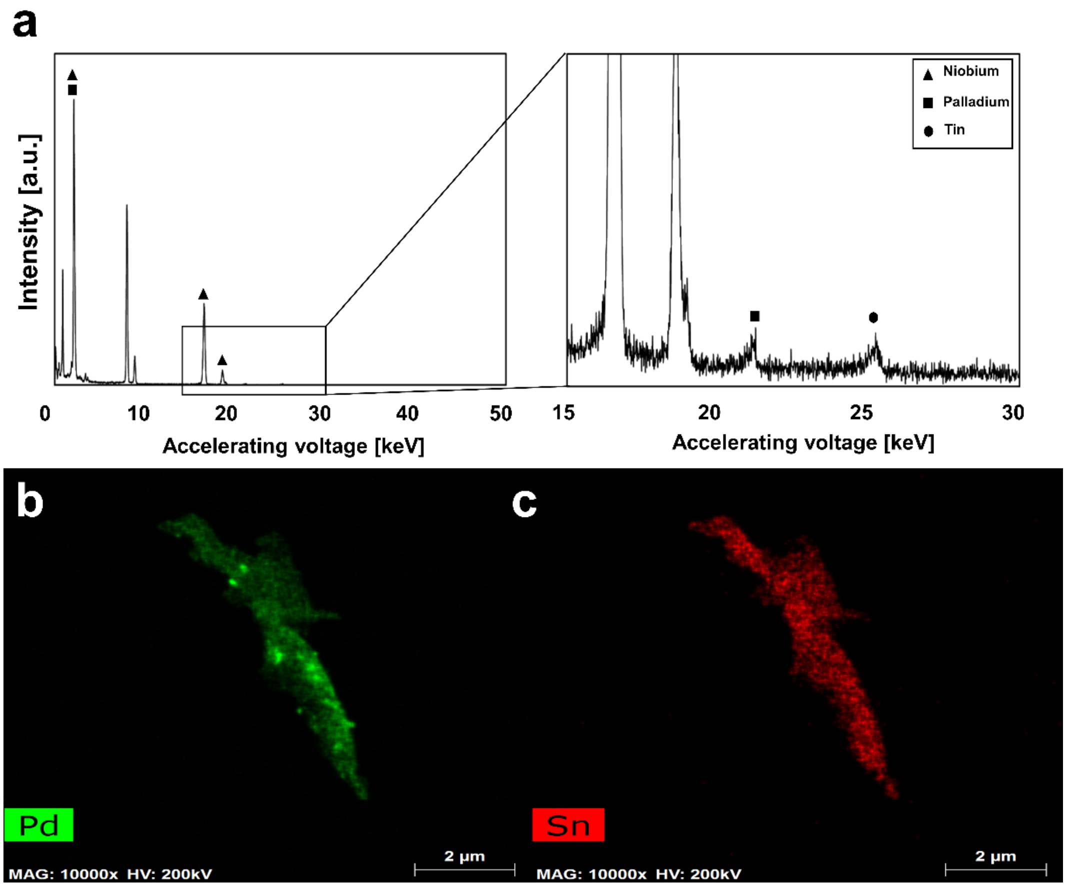

Figure S2. Sn residue of electroless plated H_2 separation membrane; (**a**) EDS spectrum of Pd(ELP), (**b**) and (**c**) EDS mapping of the sample.

Pd(ELP)Cu(SPT)/Ta Morphology Before and after Permeation Test for 10 h

The SEM images in Figure S3 show the formation of cracks on the entire surface of the membrane after operation for 10 h.

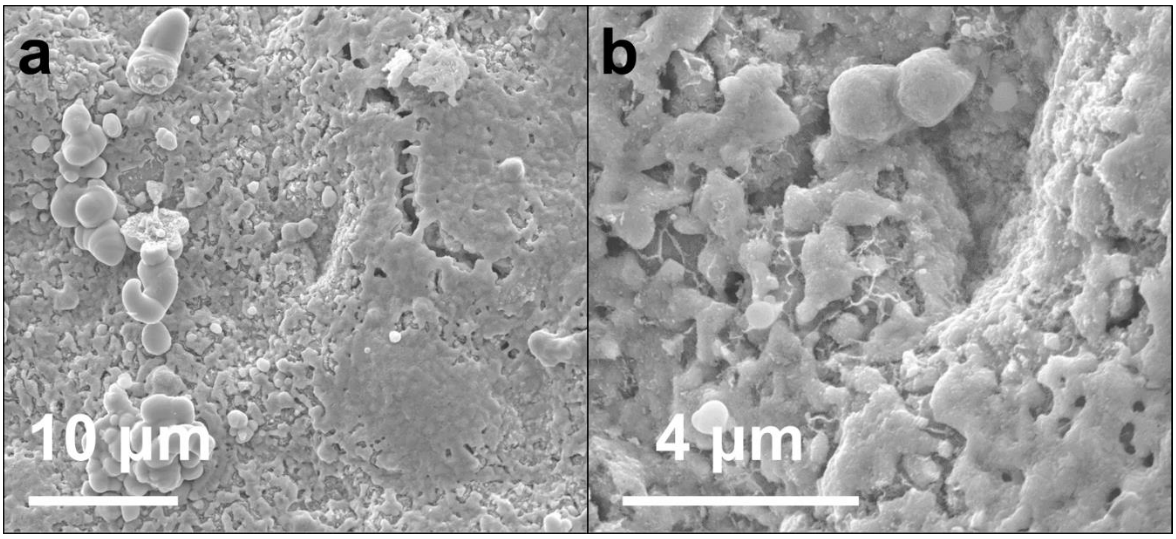

Figure S3. SEM iamge of Pd(ELP)Cu(SPT)/Ta surface after operation for 10 h at (**a**) 5,000x magnification, and (**b**) 20,000x magnification.

References

1. Wei, L.; Yu, J.; Hu, X.; Wang, R.; Huang, Y. Effects of Sn residue on the high temperature stability of the H2-permeable palladium membranes prepared by electroless plating on Al2O3 substrate after SnCl2–PdCl2 process: A case study. *Chin. J. Chem. Eng.* **2016**, *24*, 1154–1160.
